# Fabrication of All Glass Bifurcation Microfluidic Chip for Blood Plasma Separation

**DOI:** 10.3390/mi8030067

**Published:** 2017-02-24

**Authors:** Hyungjun Jang, Muhammad Refatul Haq, Jonghyun Ju, Youngkyu Kim, Seok-min Kim, Jiseok Lim

**Affiliations:** 1 School of Mechanical Engineering, Chung-Ang University, Seoul 06974, Korea; janghj@cau.ac.kr (H.J.); refat@cau.ac.kr (M.R.H.); jhju@cau.ac.kr (J.J.); kykdes@cau.ac.kr (Y.K.); 2 School of Mechanical Engineering, Yeungnam University, Gyeongsan-si, Gyeongsangbuk-do 38541, Korea

**Keywords:** glass molding, amorphous carbon mold, microfluidics, blood plasma separation, bifurcation microfluidic chip

## Abstract

An all-glass bifurcation microfluidic chip for blood plasma separation was fabricated by a cost-effective glass molding process using an amorphous carbon (AC) mold, which in turn was fabricated by the carbonization of a replicated furan precursor. To compensate for the shrinkage during AC mold fabrication, an enlarged photoresist pattern master was designed, and an AC mold with a dimensional error of 2.9% was achieved; the dimensional error of the master pattern was 1.6%. In the glass molding process, a glass microchannel plate with negligible shape errors (~1.5%) compared to AC mold was replicated. Finally, an all-glass bifurcation microfluidic chip was realized by micro drilling and thermal fusion bonding processes. A separation efficiency of 74% was obtained using the fabricated all-glass bifurcation microfluidic chip.

## 1. Introduction

Microfluidic chip technology has attracted much attention in the field of biological analysis because it decreases the amount of the sample and the time required for analysis [1,2,3,4]. Blood plasma provides plenty of vital information about health condition [5], and is one of the most widely used target fluids in microfluidic systems for disease diagnosis [6]. Blood plasma is commonly extracted using a centrifuge machine. However, the procedure is time-consuming and requires relatively large amounts of blood sample; hence, it is not suitable for use with the microfluidic chip technology. The blood plasma separation processes have been conducted on a microfluidic platform. The microfluidic methods for blood plasma separation can be categorized into two types: active and passive. The active methods use external forces such as centrifugal [7,8], acoustic [9], electric [10,11], and magnetic [12] forces, and the passive methods use hydrodynamics [13,14,15,16] or geometrical filter structures [17,18,19] for blood plasma separation. Although the centrifugal microfluidic platform has been effectively used for separating and analyzing blood plasma, it is not suitable for continuous real time blood separation and analysis processes, which are required in some medical treatments such as blood dialysis for renal insufficiency patients and cardiac surgery with cardiopulmonary bypass [20]. The bifurcation microfluidic chip [20,21,22], a passive chip using hydrodynamics, can be regarded as a suitable method for continuous blood plasma separation because it can separate blood plasma from the main blood stream and does not require any additional device for generating external force. In the bifurcation microfluidic chip, the blood plasma separation efficiency is mainly affected by the geometrical shape of the chip and the efficiency increases with increasing flow rate [23,24]. Therefore, dimensional stability at high flow rates is essential for the blood plasma separation microfluidic chip using bifurcation. In the fabrication of the bifurcation microfluidic chip, most of the researchers use a polydimethylsiloxane (PDMS) replication process because of its simplicity and cost-effectiveness. However, the PDMS microfluidic chip has some limitations regarding dimensional stability at high flow rates due to its elastic characteristics.

In this research, we fabricated a highly durable glass bifurcation microfluidic chip for blood plasma separation. To realize a glass microfluidic chip, various patterning techniques based on the material removal of glass substrate have been investigated, such as mechanical milling [25], laser ablation [26], and etching process [27]. However, these methods are time-consuming due to the high mechanical, chemical, and thermal resistances of the glass materials. As an alternative method for patterning the glass material at low cost, a glass molding process has been proposed. In this process, the glass substrate is heated up to higher than its glass transition temperature and pressed against a mold with the negative shape of the final structure. During the molding process, the cavity patterns of the mold are transferred onto the surface of the glass substrate. Since the processing temperature of glass molding (>600 °C) is much higher than that of the conventional polymer molding, the nickel mold commonly used in the micro polymer molding processes cannot be utilized for glass molding due to its low mechanical resistances at high temperatrue. Nickel alloy [28], silicon carbide [29], and amorphous carbon (AC) [30] have been used as mold materials for glass molding of microfluidic structures due to their high hot hardness. To prepare the cavity structure in these refractory materials, laser aberration was applied in the previous works [28,29,30]. However, the surface quality of the mold cavity pattern fabricated by laser aberration was not suitable for precise glass microfluidic chip applications. Furthermore, the laser aberration method is not suitable for fabricating many molds with the same design, which are required in the mass production of glass molded microfluidic devices, because it is a serial machining process and a long machining time is required due to the low material removal-rate of refractory materials and the large machining area to produce a protruding microchannel cavity. In our previous paper, we proposed a low-cost, parallel, and large-area fabrication method for an AC micro mold with high surface quality by the carbonization of a replicated furan precursor [31].

**Figure 1:**
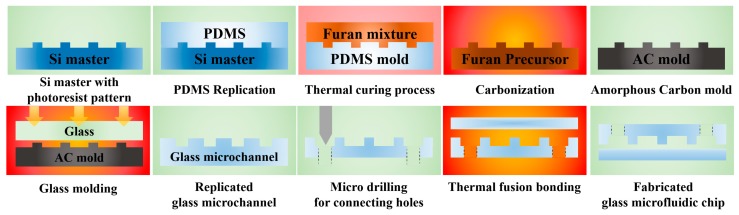
Schematic of the fabrication process for the AC mold by the carbonization of a replicated furan precursor and the all-glass bifurcation microfluidic chip by glass molding and thermal fusion bonding.

In this research, an all-glass bifurcation microfluidic chip for blood plasma separation was fabricated, using the glass molded microchannel plate prepared from an AC mold. Figure 1 shows the schematics of the steps of the fabrication process for the all-glass bifurcation microfluidic chip used in this study. Although various patterning techniques [32,33,34,35] can be applied as a master fabrication process, conventional photolithography was applied in this study. To compensate for the shape change during AC mold fabrication, an enlarged silicon master pattern was prepared. A furan precursor was obtained by thermal replication using a replicated PDMS mold from the master. After the carbonization of the furan precursor, an AC mold with microchannel cavities was obtained. Finally, the glass bifurcation microfluidic chip was obtained by implementing glass molding for the microchannel plate, micro-drilling for the inlet and outlet connecting holes, and thermal fusion bonding for sealing. To examine the feasibility of the all-glass bifurcation microfluidic chip, the performance of the blood plasma separation was evaluated.

## 2. Fabrication of the All-Glass Microfluidic Chip Using a Glass Molded Microchannel

### 2.1. Design and Fabrication of the Silicon Master

**Figure 2:**
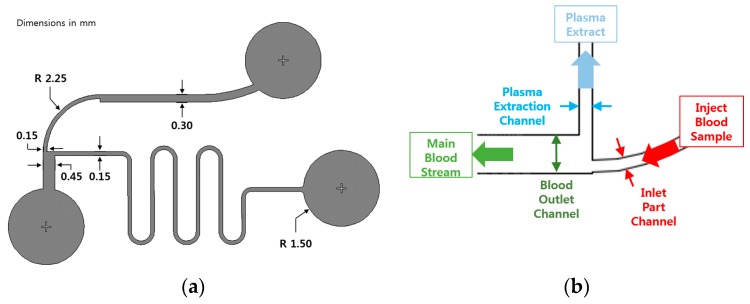
Schematics of (**a**) the designed bifurcation blood plasma separation chip and (**b**) the main separation region of the designed bifurcation blood plasma separation chip.

A bifurcation microfluidic chip for blood plasma separation was examined for the feasibility of the glass molded microfluidic chip prepared using AC mold. Figure 2a shows the design of the blood plasma separation chip. The tortuous inlet microchannel was designed to improve the separation efficiency due to the centrifugal action [21]. Figure 2b shows the schematic of the main separation region of the bifurcation microfluidic chip. The designed widths of the blood inlet, blood outlet, and plasma extraction channels were 150, 450, and 150 μm, respectively, and the height of the channel was 55 μm. The performance of the bifurcation microfluidic chip can be examined using the channel resistance ratio between the plasma extraction and the blood outlet channels. The channel resistance (*R*) can be calculated by Equation (1) [36].

(1) $$R=\;\frac{12\mu L}{\left\{1-0.63\left(\frac{h}{w}\right)\right\}}\frac{1}{h^{3}w}$$

where μ is the viscosity of the blood (0.004 Pa·s) and *L*, *w,* and *h* are the length, width, and height of the channel, respectively. The lengths of the blood outlet and plasma extraction channels were 1.5 and 26.7 mm, respectively. The calculated channel resistance ratio between the plasma extraction and blood outlet channels was 63:1, which is an acceptable value for plasma extraction [36].

In the proposed AC mold fabrication process, a significant shape change inherently occurs during carbonization due to the material decomposition [31]. In addition, some shrinkages also occurred in PDMS and Furan replication processes due to the polymerization of material. To compensate the total shrinkage occurring in the AC mold fabrication process, we conducted a preliminary experiment using the master pattern having similar microchannel structures. In the preliminary experiment, the total shape change (shrinkage) of the AC mold compared to the master pattern was ~25%. To compensate for the shape change during AC mold fabrication, an enlarged (~133%) photoresist master with a positive microchannel structure (inverse shape of the final microchannel structure, positive channel structure) was fabricated. The dimensional targets for the master pattern were 200 μm for the width of the inlet and extraction channels, 600 μm for the outlet channel, and 70 μm for the height. About 5 g of negative photoresist (PR, SU-8 3050, Microchem Co. Ltd., Westborough, MA, USA) was poured onto a 4-inch Si wafer, and spin coated at a maximum rotation speed of 3000 rpm for 25 s to achieve a PR thickness of 70 μm. The coated photoresist was prebaked on a hot plate at 95 °C for 15 min and exposed to 20 mW/cm^2^ UV light for 12 s using a mask aligner system (M-100, Prowin Co. Ltd., Daejeon, Korea). A post-exposure bake step was then conducted on a hot plate at 65 °C for 1 min and at 95 °C for 5 min. After the development process using the SU-8 developer for 8 min, the developed pattern was rinsed with isopropyl alcohol (IPA). Finally, a hard bake step was conducted on a hot plate at 100 °C for 1 h.

### 2.2. Fabrication of the AC Mold

An AC mold with microchannel cavities (positive channel structure) was fabricated by the carbonization of a replicated polymer precursor. A PDMS mold with the negative channel structure was replicated from the photoresist master pattern. An elastomer, Sylgard 184A (Dow Corning Co. Ltd., Auburn, MI, USA), was mixed with Sylgard 184B in a weight ratio of 10:1, poured on the photoresist master pattern, and cured on a hot plate at 150 °C for 1.5 h. Furan mixture—made of furan resin (Kangnam Chemical Co. Ltd., Seoul, Korea), *p*-toluenesulfonic acid (Kanto Chemical Co., Tokyo, Japan), and ethanol—was poured into the PDMS mold. To remove the air bubbles entrapped in the furan mixture during the mixing and pouring processes, a degassing process was conducted by placing the PDMS mold with the furan mixture into a vacuum chamber at room temperature for 3 h. After degassing, a two-step curing process was carried out to avoid the warpage of the replicated furan precursor. In the first curing phase, the furan mixture was cured under atmospheric conditions for five days, and in the second phase, it was cured in a convection oven for two days at ~100 °C. After curing, the furan precursor with positive microchannel structures was released from the PDMS mold and carbonized in a tube furnace at a maximum temperature of 1000 °C under nitrogen environment for five days. After carbonization, an AC mold with positive microchannel cavities was obtained [31]. Since the furan material is a carcinogen, the processes using furan was conducted in a fume hood.

### 2.3. Glass Molding of Microfluidic Channel Plate

**Figure 3:**
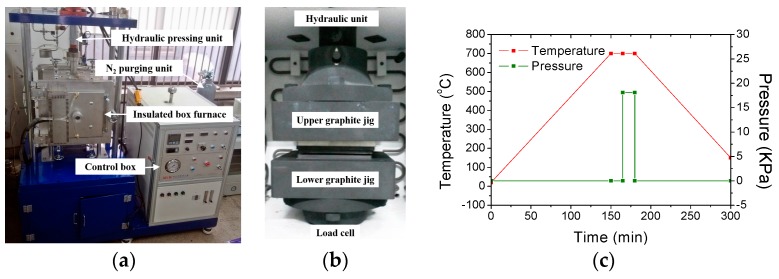
Photographs of the (**a**) constructed glass molding system; (**b**) graphite pressing jig in the box furnace; and (**c**) temperature and pressure history during the glass molding process.

**Figure 4:**
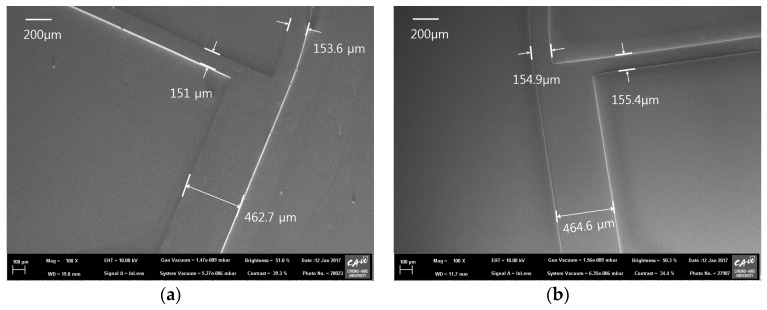
SEM images of fabricated (**a**) AC mold and (**b**) glass molded microchannel at blood separation region.

A glass molding machine, comprising an insulated box furnace fitted with carbon heaters for heating up to 1000 °C at a heating rate of 5 °C/min, a hydraulic pressing unit for applying a compression pressure of ~30 ton during the glass molding, and a nitrogen purging unit for maintaining an inert gas environment during the glass molding, was designed and constructed as shown in Figure 3a. In the box furnace, the top and bottom graphite pressing jig structures (as shown in Figure 3b) were connected to the hydraulic unit and the load cell, respectively. A soda-lime glass plate (Low-Iron Glass, JMCGLASS Co. Ltd., Seoul, Korea) with a glass transition temperature (*Tg*) of 650 °C and a softening temperature (*Ts*) of 720 °C was used as the molding material. In the glass molding process, the fabricated AC mold was diced to a size of 20 × 15 mm^2^ and placed on the bottom graphite jig. A glass plate with the same size as that of AC mold and a thickness of 3.2 mm was loaded onto the AC mold. To prevent the oxidization of the AC mold during the glass molding process, the box furnace was evacuated and purged with nitrogen gas. The glass molding process was divided into four stages: heating, holding, pressing, and cooling. Figure 3c shows the temperature and pressure history during the entire glass molding process. In the heating stage, the temperature of the furnace (ambient temperature of the AC mold and the glass plate) was increased up to the molding temperature of 700 °C. In the holding stage, the temperature was maintained for 15 min to achieve a stable temperature in the furnace (uniform temperature distribution between the AC mold and the glass plate). In the pressing stage, a compression pressure of 18 kPa was applied to the stack of the AC mold and glass plate for 15 min. In the cooling stage, the pressure was released and natural cooling was carried out. When the furnace temperature was reduced to the room temperature, the box furnace was opened and the replicated glass plate was detached from the AC mold. Figure 4 shows the SEM images of the fabricated (a) AC mold and (b) glass molded microchannel at blood separation region.

### 2.4. Preparation of the All-Glass Bifurcation Blood Plasma Separation Chip

To develop the blood plasma separation chip, three connection holes for blood in-flow, blood out-flow, and plasma out-flow were machined on the glass molded microchannel plate by the micro drilling process. A drill press (MM-180s, MANIX Co. Ltd., Pyeongtaek, Korea) with a carbide drill tool (φ1.5 mm, HAM precision Co. Ltd., Pewaukee, WI, USA) was used at a rotation speed of 2000 rpm. After drilling, the glass plate with microchannels was sufficiently cleaned by an ultrasonic cleaner using acetone and ethanol for 5 min each, because any particle or dust on the plate can cause failure of the subsequent glass sealing process. Finally, a planar glass plate was bonded onto the fabricated glass microchannel plate by a thermal fusion bonding process at a temperature of 630 °C and a pressure of 3 kPa for 3 h using the constructed glass molding system.

## 3. Analysis of the Geometrical Properties in Each Fabrication Step

**Figure 5:**
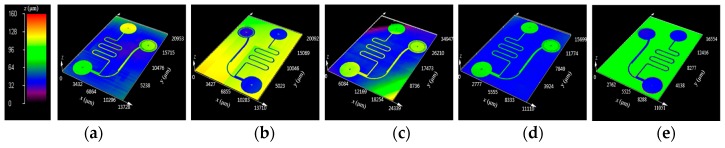
3D microscope images of the (**a**) master pattern on Si wafer; (**b**) polydimethylsiloxane (PDMS) mold; (**c**) furan precursor; (**d**) AC mold; and (**e**) replicated glass microfluidic structure.

**Figure 6:**
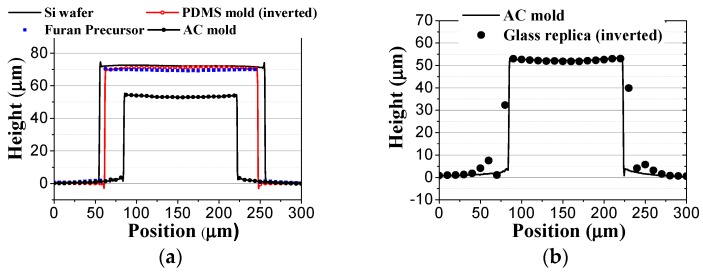
Comparison of cross-sectional surface profiles of the (**a**) silicon master, furan precursor, and AC mold; and (**b**) AC mold and replicated glass microfluidic structure.

To examine the geometrical properties of the fabricated samples, the surface profiles of each microfluidic channel structure were measured by a 3-dimensional (3D) confocal microscope (OLS-4100, Olympus Co. Ltd., Tokyo, Japan). Figure 5 shows the 3D surface profiles of the fabricated (a) silicon master; (b) PDMS mold; (c) furan precursor; (d) AC mold; and (e) molded glass microfluidic plate, obtained from the confocal microscopy results. It is clearly seen that the positive and negative microfluidic channel structures were uniformly transferred in each fabrication step. Figure 6 shows the comparisons of the cross-sectional surface profiles of the inlet microchannel (a) between the silicon master, PDMS mold (inverted), furan precursor, and AC mold, and (b) between the AC mold and the glass replica (inverted). Figure 6a shows that a small shape change (shrinkage) occurred during the PDMS and furan precursor replication processes due to polymerization, and a significant shape change took place during the AC mold fabrication because of material decomposition during carbonization. Although an inherent substantial shape change occurred during the AC mold fabrication process, the shape change during the glass molding process is negligible, as shown in Figure 6b.

For the quantitative analysis of the changes in the geometrical dimensions after each fabrication step, for a glass molded microfluidic chip with AC mold, the measured widths and heights of microchannels in each sample are summarized in Table 1. Although the target values of the silicon master pattern were 200, 600, 200, and 70 μm for the inlet width, outlet width, extraction width, and height, respectively, the fabricated photoresist master pattern had a dimensional error of 1.6% (average) due to the experimental errors in the spin coating and photolithography processes. The shape difference between the silicon master and the replicated furan precursor was 4.2% (dimensional change/dimension of master, average), and it might have been caused by the shrinkage during the polymerization of PDMS and furan materials. The shrinkage ratio due to the material decomposition in carbonization, which is the dimensional change ratio between the AC mold and the furan precursor (dimensional change/dimension of furan precursor), was ~22% (average). Therefore, the total shrinkage ratio between the AC mold and the silicon master (dimensional change/dimension of master) was 25.2% (average), which is almost the same as the values used for compensating the enlargement of the master pattern size. The standard deviation of the shrinkage rate was ~1.05% in the carbonization process and 1.42% in whole AC fabrication process. In this study, the designed values for the widths of the inlet, outlet, and extraction, and the height of the bifurcation fluidic chip were 150, 450, 150, and 55 μm, respectively. The average shape error between the design and the fabricated AC mold was 2.9%. Since the shape error in the conventional PDMS replication process, widely used in the microfluidic chip fabrication, was ~2%, the dimensional error of ~3% in the AC mold fabrication with our shrinkage compensation method is acceptable for the mold fabrication method, for microfluidic chip applications. To examine the repeatability of the proposed AC mold fabrication process, we fabricated five AC molds using the same master pattern. The average shrinkage ratio in whole AC mold fabrication process were 24.6%~25.7%. Although ~1% of shrinkage rate variation was existing in the repeated experiment, we believe that is acceptable in microfabrication process.

In the glass molding process, the widths and heights of microchannels in the glass molded plate were slightly greater than those of the AC mold (~1.5%). This difference might have been caused by the measurement errors of the confocal microscope while measuring the positive and negative shapes. Therefore, the shape change during the glass molding process can be neglected. Although the fabricated glass microfluidic channel has some dimensional errors compared to the designed values, the channel resistance ratio between the plasma extraction and the blood outlet channels calculated using the measured data was ~65:1, which is similar to the value obtained using the designed data.

We also measured the surface roughness of the fabricated samples at the bottom location of the microchannel. The measured root mean square (RMS) surface roughness values were 8, 34, 30, 12, and 8 nm on the silicon master, PDMS mold, furan precursor, AC mold, and molded glass plate samples, respectively. Although the surface roughness increased for the PDMS and the furan precursor due to the chain size of the polymer materials, it decreased during carbonization owing to the inherent shrinkage. In the glass molding process, the surface roughness also improved probably due to the surface energy of the softened glass material. It is clearly seen that the proposed AC mold fabrication process can provide a mold for glass molding with superior surface quality, which is difficult to obtain using the previous laser machined molds.

## 4. Feasibility Analysis of the All-Glass Bifurcation Chip for Blood Plasma Separation

**Figure 7:**
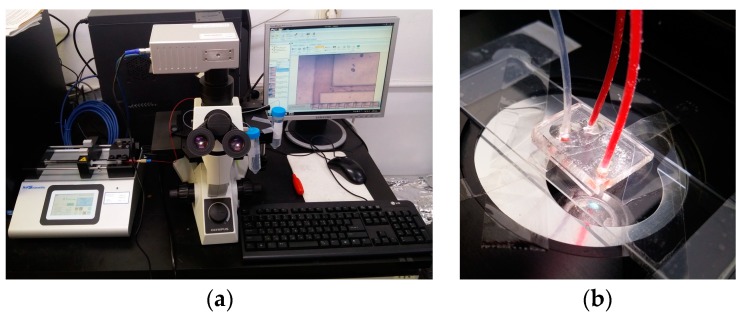
Photographed images of the (**a**) experimental setup for blood plasma separation and (**b**) fabricated all-glass bifurcation microfluidic chip during blood plasma separation.

**Figure 8:**
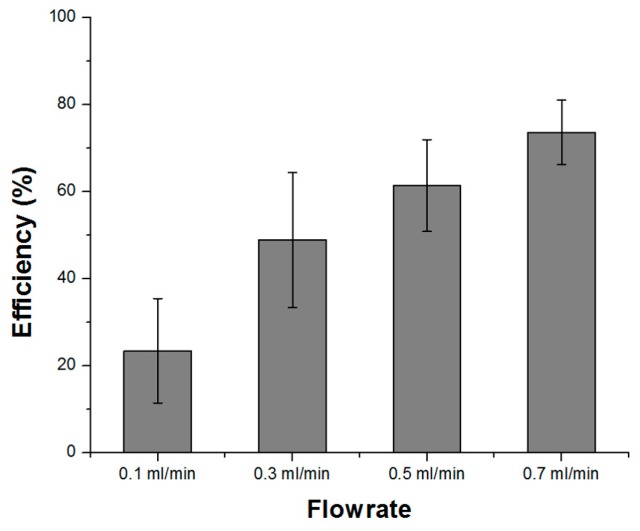
Blood plasma separation efficiency with various flow rates in the fabricated all-glass microfluidic chip with diluted and defibrinated sheep blood.

To examine the feasibility of the fabricated glass bifurcation microfluidic chip, we prepared a setup for blood plasma separation composed of an inverted microscope (CKX-41, Olympus Co., Tokyo, Japan) and a precision syringe pump (Legato 200, KD Scientific., Ringoes, NJ, USA). For the blood plasma separation experiment, diluted defibrinated sheep blood (MBcell, Los Angeles, CA, USA) in phosphate-buffered saline (PBS) with a hematocrit level of 25% [20] was infused into the glass bifurcation microfluidic chip using the precision syringe pump at flow rates of 0.1, 0.3, 0.5, and 0.7 mL/min. Figure 7a shows the experimental setup for imaging the blood plasma separation and Figure 7b shows the fabricated glass bifurcation microfluidic device with tubing during the separation process. Three Teflon tubes were connected to the micro-drilled inlet and outlet holes, and sealed using UV curable bonding material. To examine the effects of flow rates on blood plasma separation, the number of red blood cells in the inlet channel and the plasma extraction channels were measured using a hemacytometer, and the blood plasma separation efficiency (*E*) was calculated by Equation (2).

(2) $$E\;=\;\frac{N_{inlet}-N_{plasma}}{N_{inlet}}\;\times \;100\;\left(\%\right)$$

where *N_inlet_* and *N_plasma_* were the measured number of red blood cells in the inlet channel (initial blood sample) and the plasma extraction channels (sample obtained from the tube attached to the plasma extraction reservoir), respectively. We fabricated five glass blood plasma separation chips and measured the blood plasma separation efficiencies twice at a flow rate of 0.1~0.7 mL/min for each chip. After the measurements with fixed flow rates, the chips were cleaned with a PBS solution for 3 min. Figure 8 shows the effects of the flow rate on the blood plasma separation efficiency. It was noted that the separation efficiency improved with increasing flow rate, and an efficiency of 74% was obtained at a flow rate of 0.7 mL/min. The relatively large variation in the measurement results might be mainly due to the large uncertainty in the blood cell measurement process using a hemacytometer [37].

## 5. Conclusions

A durable all-glass bifurcation microfluidic chip was fabricated using a glass molded microchannel plate. An AC mold with microchannel cavities was fabricated by the carbonization of a replicated furan precursor, which is a low-cost and large-area patterning process for achieving high surface quality. During the AC mold fabrication process, a large and inevitable shrinkage (~22%) occurred in carbonization process due to the thermal decomposition process, and ~4% of shrinkage also occurred in PMDS and furan replication due to polymerization. To compensate the total shrinkage (~25%) during the fabrication of the AC mold, an enlarged photoresist master pattern was fabricated by photolithography on a silicon wafer. A PDMS mold was replicated from the silicon master and a furan precursor was replicated from the PDMS mold by the thermal curing process. The AC mold was obtained by the carbonization of the furan precursor. The shape difference between the AC mold and the silicon master was ~25.2%, which is similar to the value used for the compensation process. Although an inherent large shrinkage occurs in the AC mold fabrication process, the dimensional error between the designed value and the measured value from the fabricated AC mold was 2.9%, which is acceptable in a microfluidic device. Furthermore, almost half of the dimensional error (1.6%) occurred during the photolithography process. It clearly shows that the large amount of shrinkage occurring in the AC mold fabrication process is predictable and can be compensated. Finally, a glass microchannel plate with high fidelity was replicated using the glass molding system, and a flat glass plate was bonded to the replicated glass microchannel plate using the thermal fusion bonding method. The long cycle time issues of glass molding and thermal fusion bonding in this research are mainly due to our stand alone type glass molding system, and it can be solved by using a progressive type glass molding system [38]. Thus, we fabricated an all-glass bifurcation microfluidic chip using the glass molded microchannel plate and measured the blood plasma separation efficiency of 74% in the fabricated chip at a flow rate of 0.7 mL/min. Although the applied flow rate and the achieved plasma separation efficiency were not higher than those reported using the conventional PDMS chip because of the limitation of our injection system, it shows that the feasibility of the proposed all-glass chip fabrication process for microfluidics application. The application of the developed device to the whole blood plasma separation with high pressure injection system and real-time monitoring of the contents in the separated plasma using Raman analysis is the subject of our ongoing research.

## Figures and Tables

**Table 1:** Summary of the measured widths and heights of the microchannels for each fabrication step.

Samples	Inlet Channel		Outlet Channel		Extraction Channel	
	Width (μm)	Height (μm)	Width (μm)	Height (μm)	Width (μm)	Height (μm)
Silicon master	204.5	71.4	600.4	71.9	200.3	71.6
polydimethylsiloxane (PDMS) mold	202.5	71.0	584.9	71.7	190.6	71.1
Furan precursor	196.4	67.8	585.9	68.2	190.8	68.9
amorphous carbon (AC) mold	153.6	51.6	462.7	53.7	151.0	53.4
Glass replica	154.9	52.9	464.6	54.4	155.4	53.9
